# Bioactive Compounds and Health-Promoting Properties of Elephant Apple (*Dillenia indica* L.): A Comprehensive Review

**DOI:** 10.3390/foods12162993

**Published:** 2023-08-08

**Authors:** Deepanka Saikia, Radhakrishnan Kesavan, Baskaran Stephen Inbaraj, Praveen Kumar Dikkala, Prakash Kumar Nayak, Kandi Sridhar

**Affiliations:** 1 Department of Agricultural Engineering, Centurion University of Technology and Management, Paralakhemundi 761211, Odisha, India; 2 Department of Food Engineering & Technology, Central Institute of Technology Kokrajhar, Kokrajhar 783370, Assam, India; 3 Department of Food Science, Fu Jen Catholic University, New Taipei City 242062, Taiwan; 4 School of Food Technology, Jawaharlal Nehru Technological University Kakinada, Kakinada 533003, Andhra Pradesh, India; dikkalapraveenkumar@gmail.com; 5 Department of Food Technology, Karpagam Academy of Higher Education (Deemed to be University), Coimbatore 641021, Tamil Nadu, India

**Keywords:** elephant apple, bioactive compound, herbal medicine, phenolic compound, biological activity

## Abstract

Elephant apple (*Dillenia indica* L.) grows wild in Southeast Asia’s forests, including in China, India, Nepal, Bangladesh, and Sri Lanka. Elephant apples are considered essential fruit crops because of their high nutritional value, which includes high levels of vitamin C, carbohydrates, fats, fibre, protein, minerals, and fatty acids. It is important to understand the nutritional value and health benefits of elephant apples in order to increase fruit intake in people’s daily diets. The present review paper focuses on elephant apple’s phytochemistry, bioactive compounds, therapeutic value, and medicinal capabilities for designing and developing a wide range of food formulations. Proteins, minerals, fats, crude fibre, carbohydrates, vitamin C, tannins, malic acid, and glucose are abundant in the leaves, bark, and fruit of the elephant apple. In addition to nutritional components, many phytochemicals found in elephant apples have been identified as bioactive compounds with a broad range of biological activities, the most prominent of which are antioxidant, anticancer, antidiabetic, and anti-inflammatory properties. Overall, elephant apple is a rich, natural source of bioactive compounds with potential applications in the production of value-added foods and nutraceuticals for disease prevention and management.

## 1. Introduction

India has over 45,000 different species of plants [1] and is among the 12 worldwide biodiversity centres [2]. Many plant species that are native to different forest areas of India are widely used by tribal and folk societies; however, the pharmacognostic and phytopharmacological importance of these plants is still unknown and remains inconclusive as they are seldom studied. Among these, there are a few plants of the Dilleniaceae family that have been reported to possess high therapeutic value, but there is still very little scientific understanding of Dilleniaceae cultivars. For instance, the genus *Dillenia* consists of 60 varieties, in which *D. pentagyna*, *D. indica*, *D. alata*, *D. papuana*, *D. suffruticosa*, *D. excelsa*, *D. ovata*, *D. serrata*, and *D. phllipinensis*, etc., are documented to have strong therapeutic values; two of these plants are only known to grow in India: *D. pentagyna* and *D. indica* [3]. Due to the presence of strong therapeutic abilities, plant parts such as fruits, leaves, and bark have received much attention as folk medicine. Moreover, many tribal and folk societies that are native to different areas prefer to ingest the raw fruits of *D. indica* and *D. pentagyna* [4,5,6].

**Figure 1:**
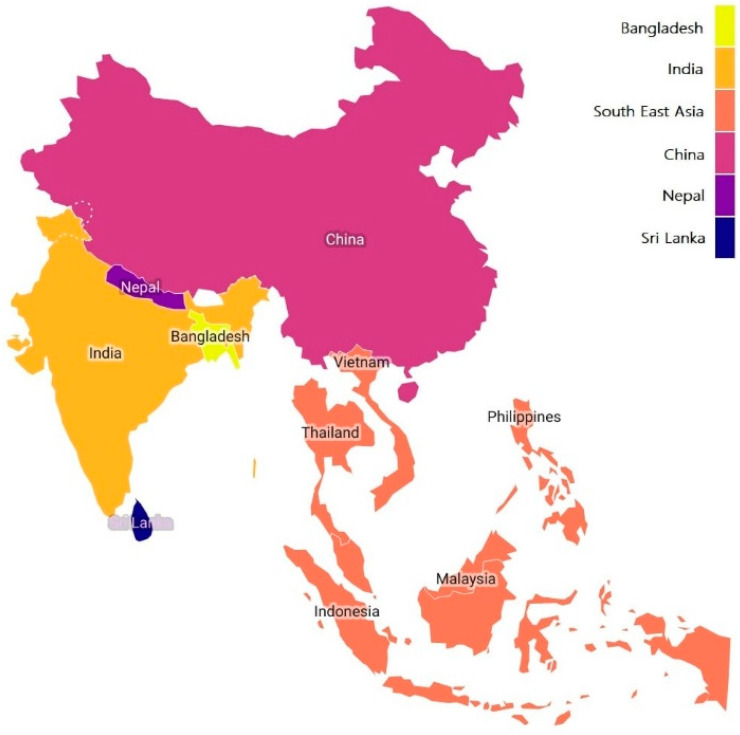
Production of elephant apple.

Elephant apple (*Dillenia indica* L.) is a tree native to Southeast Asia, including India, China, Sri Lanka, Nepal, and Bangladesh (Figure 1). The elephant apple tree is a small- to medium-sized evergreen shrub that can grow up to 15 m tall. In particular, it grows extensively in the moist and evergreen forests of Northeastern India [7,8,9]. It grows in the sub-Himalayan region between Garhwal and Assam, Arunachal Pradesh, Tripura, Manipur, West Bengal, Bihar, and Orissa along with Central and Southern India, and in forests at low and medium altitudes in Nepal, Bangladesh, and Sri Lanka [10,11,12]. It was introduced in 1908 in the Philippines and has now spread across the whole continent [13].

**Figure 2:**
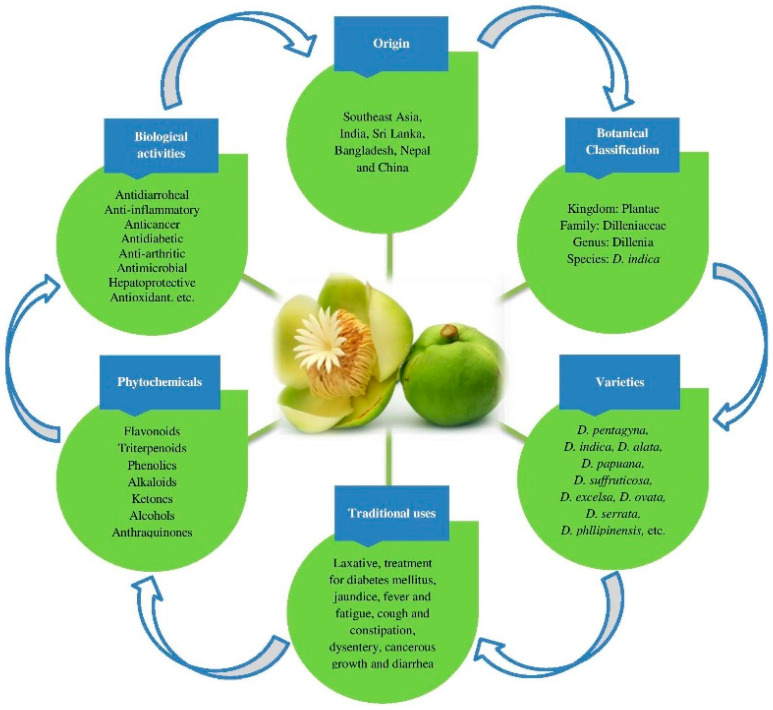
Principal generalities of elephant apple fruit.

Elephant apple’s leaves, bark, and fruits contain a large number of flavonoids and triterpenoids (lupene-type), along with phenolics, ketones, phytosteroids, alcohols, and anthraquinones. Diverse forms of active substances, such as betulin, myricetin, dillenetin, betulinic acid, β-sitosterol, kaempferol, stigmasterol, quercetin, and rhamnetin, contribute to the range of phytochemicals of therapeutic importance in elephant apple. The key chemical constituents are betulin and betulinic acid (lupene-type triterpenoids), which have a wide range of therapeutic properties [12,14]. The general aspects and characteristics of elephant apple that are exploited for common and traditional uses are shown in Figure 2.

Several scientific and clinical investigations have been conducted over the years to determine the beneficial effects of the consumption of elephant apple. Several studies have reported the functional activity of elephant apple, including free radical scavenging, haematoprotective, antidiabetic, anti-inflammatory, and antimicrobial properties [14,15,16,17,18,19]. Nevertheless, these investigations were highly restricted to the individual functional activities of elephant apple, and there is a paucity of evidence to provide a systematic overview on the functional activities of elephant apple. Thus, a review dealing with the major functional properties of elephant apple is necessary for the potential application of elephant apple in the food industry.

Therefore, this paper attempts to review the current trends in the bioactive components of elephant apple and its biological role in treating and preventing life-threatening diseases. This critical review will provide deeper insights for an in-depth exploration of the phytochemistry and therapeutic importance of elephant apple to design and develop a wide variety of food formulations.

## 2. Classification and Chemistry

**Figure 3:**
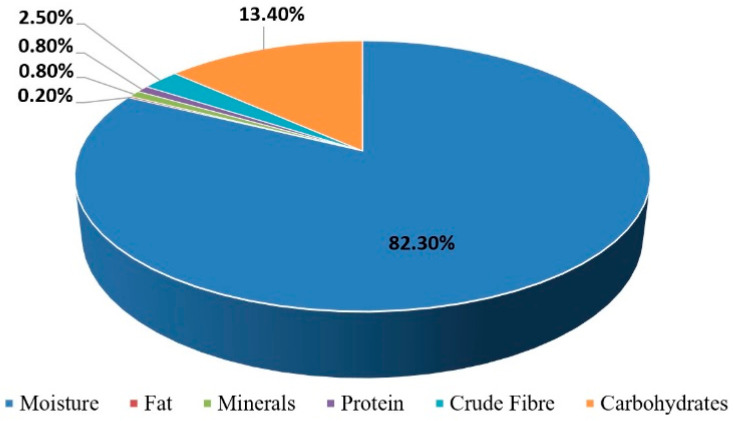
Approximate composition of edible portion of elephant apple.

Elephant apple *(Dillenia indica)* belongs to the family Dilleniaceae. Previous studies have highlighted the presence of primary and secondary metabolites in elephant apple. The primary metabolites, like proteins, minerals, fats, crude fibre, and carbohydrates, that are present in elephant apples are shown in Figure 3 [20]; they are mainly responsible for cell maintenance. Compared to primary metabolites, the chemical structure and properties of secondary metabolites are complex and responsible for plant protection [21]. Using different solvents, the fruits were found to contain flavonoids, phytochemicals, steroids, triterpenoids, and alkaloids, along with tannins [22].

The bark of elephant apple is found to contain 10% tannin, triterpenoids, like betunaldehyde, lupeol, and betulinic acid, as well as flavonoids such as rhamnetin, dillenetin, dihydro-isorhamnetin, myricetin, quercetin derivatives, naringenin, and kaempferol glucoside [23,24]. The presence of betulin, betulinic acid, lupeol, betulinaldehyde, sitosterol, and flavonol myricetin in elephant apple bark has also been successfully identified [25].

Elephant apple is a good source of ash, acid-insoluble ash, and water-soluble ash. A study that focused on phytochemicals indicated that the plant contains terpenoids, tannins, glycosides, alkaloids, etc. [26]. Moreover, elephant apple seeds also contain fixed oils, sterols, colours, saponins, glycosides, proteins, sugars, free amino acids, tannins, and free acids [27].

Secondary metabolites are natural products that provide bioactive compounds to the plant. Flavonoids, terpenoids, and alkaloids are essential secondary metabolites that function as free radical scavengers, insect pollinators, and phytoalexins. Others may be active in antimicrobial action or cellular signalling. Flavonoids, terpenoids, alkaloids, and other secondary metabolites are reported to be found in elephant apple and are classified according to their chemical structure, as shown in tables below. Flavonoids and triterpenoids (lupine-type) are the essential chemical compounds isolated from elephant apple. The richness of phytochemistry in elephant apple is also enhanced by several other secondary metabolites, including phytosteroids, ionone, anthraquinone, diterpene, phenolics, ketones, and alcohols. A comprehensive study of phytoconstituents in elephant apple reported the presence of 60 compounds, which are discussed in this review and can also lead to additional investigations for finding new chemical compounds. Some of the major classes and miscellaneous compounds are highlighted in the following sections.

### 2.1. Phenolic Compounds

Phenolic compounds, such as flavonoids, allied phenolic, and polyphenolic compounds, are among the most common secondary metabolites found in plants. Flavonoids are polyphenolic compounds that consist of a wide group of plant secondary metabolites [28]. These are classified into flavonols, flavanones, flavones, isoflavone, anthocyanin, catechin, and chalcones according to their molecular structures. The reported health-promoting benefits of such natural products as antioxidants are very significant [22,29,30]. So far, there have been over 9000 recorded flavonoids [31]. Elephant apple is found to contain different classes of flavonoids and phenolic compounds according to their chemical structure.

Phenolic compounds are natural derivatives that develop biogenetically from the shikimate/phenylpropanoid process, which produces phenylpropanoids directly. It has one or more hydroxyl substituents on its aromatic ring [32,33]. These compounds may play a significant role in plant growth and reproduction, defending against pests and pathogens [30]. In addition, these compounds further contribute to fruit colour and sensory attributes [21]. Methanol extract obtained from the fruit of elephant apple is reported to contain 34% of the total phenolics and polysaccharides, such as arabinogalactans [34].

The ethyl acetate fraction of elephant apple bark was analysed using paper spray ionisation mass spectroscopy [35]. It reported the presence of three phenolic acids, namely 2-*O*-caffeoylhydroxycitric acid, 3,5-dihydroxy-4-methoxybenzoic acid, and 2-caffeoylisocitric acid, along with five flavonoids: 6,7,3′-trihydroxy-2′,4′-dimethoxyisoflavan (bryaflavan), amoradinin, 5,7-dimethoxyapigenin, formononetin 7-glucoronide, and mallotus B (isoallorottlerin). Moreover, an ethanolic extract of elephant apple bark, wood, and pericarp was found to contain flavonoids such as quercetin, isorhamnetin, dillenetin, naringenin, sitosterol, kaempferol, dihydrokaempferol, and gallic acid [36]. In addition, the ethanolic extract of the bark of elephant apple was identified to contain two flavonoids, kaempferol glucoside and quercetin derivatives, alongside triterpenoids [37]. The fruit of elephant apple was also found to contain cycloartenone and n-hentriacontanol. Fleshy sepals of the fruit were rich in glucose, malic acid, and arabinogalactans. The bark and wood of elephant apple contained myricetin, dihydroisorhamnetin, hydroxylactone, and glucosides [38].

Furthermore, fresh elephant apple leaves were analysed through phytochemical experiments using acid-hydrolysis, which resulted in the identification of dihydrokaempferide and naringenin 7-glucosides, as well as the formation of 10 flavonols [39]. The methanolic extract of elephant apple leaves revealed a wide range of bioactive phytochemicals, indicating its potential as a source of therapeutically effective compounds (Table 1) [40]. Flavonoids; including quercetin and kaemferol; amino acids, like glutamic acid; and sterols, such as cholesterol, were found in elephant apple leaves. Additionally, unidentified compounds were observed during TLC chromatography. The involvement of flavonoids in various biological activities, including antibacterial, anti-inflammatory, anti-allergic, antiviral, anti-tumour, and neurodegenerative disease treatments, has been well documented. They also exhibit cytotoxic effects and play roles in preventing lipid peroxidation, capillary permeability, and platelet aggregation. Furthermore, flavonoids act as potent antioxidants, free radical scavengers, and divalent cation chelators due to their strong hydrogen-donating ability [41].

### 2.2. Terpenoids

Triterpenoids (and steroids) are among the largest higher terpenoid families, containing over 1500 compounds and about 40 skeletal forms. Because of the links between cholesterol and steroids and heart disease and human physiology, sterols (generally from C27 to C30) are treated differently in medicine than terpenes. However, sterols, which are not triterpenes, may be considered a subset of 30-carbon higher terpenoids [46]. Phytosterols, such as stigmasterol, campesterol, and sitosterol, are a large class of organic compounds, known as sterols or steroidal alcohols, found in plants. In addition, phytosterols have been shown in clinical trials to block cholesterol absorption sites in the human intestine, lowering cholesterol levels [47]. Elephant apple leaves are found to contain flavonoids, tannins, triterpenoids, and steroids; petroleum extract of elephant apple leaves is found to contain sitosterol, cycloartenone, betulin, and n-hentriacontanol, while chloroform extract is found to contain betulinic acid [48]. After fractionation with n-hexane and chloroform, methanolic extracts of the leaves form compounds like β-sitosterol, betulinic acid, dillenetin, and stigmasterol [43].

Betulinic acid was isolated and quantified from various fractions (n-butanol, ethyl acetate, methanol, and water) using validated HPLC. The ethyl acetate fraction showed the highest level (97.99 ± 7.61 mg/g fraction) [49]. Betulinic acid was found in significant amounts in the pericarp, bark, timber [36], and fruit [49] of elephant apple. The methanolic extract of elephant apple contains lupeol, betulin, and β-sitosterol (Table 2) [50]. Upon partitioning with n-hexane, chromatographic column isolation of the methanolic extracts from elephant apple stems yielded four compounds: stigmasterol, betulinic acid, betunaldehyde, and lupeol [15]. Betulin, β-sitosterol, and betulinic acid were identified in all parts of elephant apple in the analysis above. Additionally, a new hydroxylactone, classified as 3β-hydroxylupane-13β-28-lactone, was extracted and characterised based on spectroscopy findings and positive Liebermann–Burchard and Zimmerman tests [25]. Furthermore, the ethyl acetate fraction of elephant apple bark reported the discovery of a new triterpenoid, nutriacholic acid, using paper spray ionisation mass spectroscopy [35].

Terpenoids, including triterpenoids and steroids, are associated with various biological behaviours. Functionally, they play a significant role in cell physiology, and modern omics approaches are being used to isolate and identify novel terpenoid compounds for potential industrial applications. Elephant apple, a plant from the Dilleniaceae family, contains different classes of terpenoids (triterpenoids and steroids), which are listed in Table 2 along with their chemical structure.

### 2.3. Alkaloids

The presence of a nitrogen atom in a heterocyclic ring unites alkaloids, a complex group of low-molecular-weight compounds. They are classified based on the composition of their carbon skeletons. Alkaloids are mainly derived from amino acids and are present in around 20% of plant species [53].

The ethyl acetate fraction of elephant apple bark using paper spray ionisation mass spectroscopy reported twelve alkaloids, such as hydroxymethylserine, 5-acetyl-2,4-dimethylthiazole, γ-Aminobutyric acid (GABA), 4-methylthiazole-5-propionic acid, N-isopropylhydrazinecarboxamide, dialanine, triethanolamine, 1,3-bis(carbamoylcarbamoyl)urea (Carbonyldibiuret), 2-(glucosyloxy) isobutyraldoxime, L-α-Aminosuberic acid, linamarin, and N-acetyl-3,5,11,18-tetrahydroxyoctadecyl-2-amine [35]. These compounds are listed in Table 3 according to their chemical structure.

Alkaloids are secondary metabolites with several therapeutic and pharmacological impacts. Because of their plausible bioactivity, around 20,000 alkaloids have been used as pharmaceuticals, stimulants, narcotics, and poisons. Certain alkaloids have been confirmed to be potentially toxic when consumed in large quantities. Many experiments have recently been carried out to classify alkaloids in underutilised fruits and vegetables. Furthermore, alkaloid-containing compound biosynthesis, aggregation, genomics, and metabolomics have also all been extensively studied [54].

### 2.4. Miscellaneous Compounds

Elephant apple contains many other compounds besides flavonoids, triterpenoids, and alkaloids, including chromane, dicarboxylic acid, fatty acid, anthraquinone, ketones, alcohols, and ethyl maltol, as well as Na and K adducts of glucose. These compounds were collected from the plants’ twigs, fruits, bark, and leaves. Table 4 lists these compounds in order of their chemical structure.

## 3. Major Biological Activities of Elephant Apple

**Figure 4:**
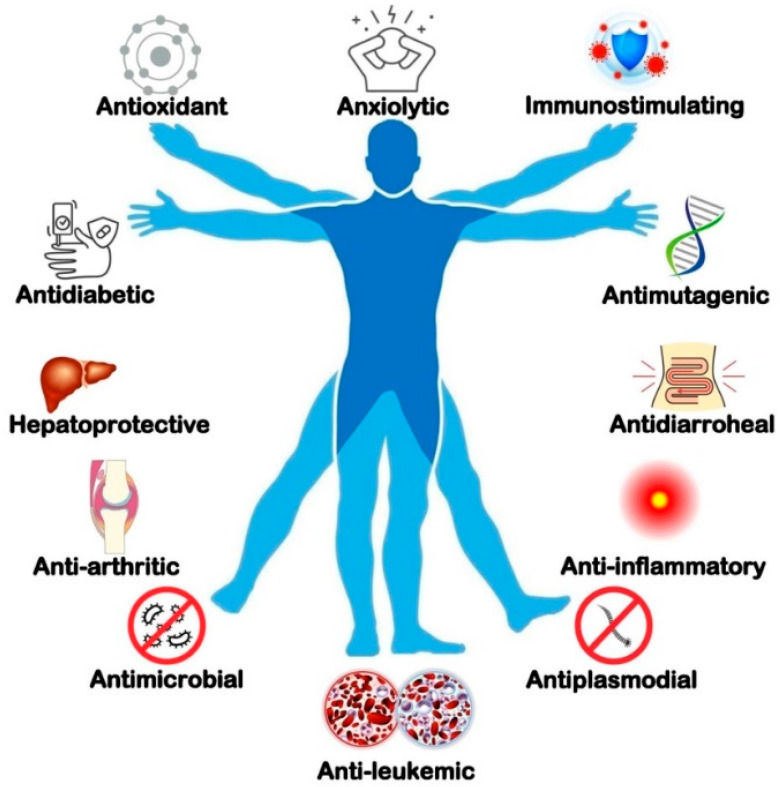
Biological activities of elephant apple (fruit, bark, and leaf) extracts.

Although many pharmacological investigations have been carried out on elephant apple, still there has been slow progress on the utilisation and exploitation of elephant apple bioactive compounds. These properties of the different parts of elephant apple have been extensively studied for the antidiarrhoeal, anti-inflammatory, anticancer, antidiabetic, anti-arthritic, antimicrobial, antioxidant, analgesic, antimutagenic, antiplasmodial, anthelmintic, anxiolytic, hepatoprotective, and immunostimulating activities (Figure 4). The important biological activities are elaborately discussed in the following Section 3.1, Section 3.2, Section 3.3, Section 3.4, Section 3.5, Section 3.6, Section 3.7, Section 3.8, Section 3.9, Section 3.10, Section 3.11, Section 3.12, Section 3.13, Section 3.14. A summary of the findings of these studies discussed below is presented in Table 5.

### 3.1. Antioxidant Activity

Antioxidants are compounds that help the body resist free radicals. Although oxygen is required for human survival, it can cause cell damage when some chemical reactions produce oxygen free radicals. These radicals are associated with the onset of illnesses, including cancer, arteriosclerosis, and ageing. Antioxidants, which can be found in both nature and the human body, guard against oxygen free radicals [57]. The health benefits of eating the fruits of elephant apple (raw or processed) and their plant parts have raised concern due to the presence of antioxidants that have defence mechanisms. Elephant apple contains phenolics, flavonoids, tannins, and terpenoids, which are major antioxidants or free radical scavenging compounds [58]. In one piece of research, elephant apple fruit was extracted with methanol, ethyl acetate, and water to determine the total phenolic content and antioxidant activity using in vitro antioxidant power models such as the phosphomolybdenum process, β-carotene/linoleate model system, and radical scavenging activity using the α, α-diphenyl-b-picrylhydrazyl (DPPH) method.

The total phenolic contents of the fruit extracts were reported to be particularly high in methanol extracts (34.14% tannic acid equivalents, *w*/*w*), followed by ethyl acetate extracts (9.37%) and water extracts (1.41%).

In the same study, the antioxidant capacity of the extracts was measured in terms of ascorbic acid equivalents (µmole/g of the extract). The free radical scavenging activities were determined as percentages (%) using the DPPH method. The antioxidant activity of the extracts, compared to synthetic antioxidant butylated hydroxyanisole, was assessed using the β-carotene/linoleate model system (% inhibition of bleaching of β-carotene) at concentrations of approximately 9.011 µmole/g and 18.022 µmole/g, respectively. Among the different extracts tested, the highest antioxidant activity was observed in methanol extracts, followed by ethyl acetate and water extracts. The findings indicate that the antioxidant properties of the extract are related to its phenolic content, indicating that fruit is a good source of antioxidants [34]. It is important to note that the isolated compound, proanthocyanidin, from fresh fruit has significant antioxidant activity, with a strong oxygen radical scavenging capacity of 1.06 × 10^4^ mol TE/g and a ferric-reducing antioxidant potential of 2320 mol Fe (II)/g [59].

Oxidative stress is a key factor in the onset and persistence of a wide range of acute and chronic medical conditions. As a result, antioxidants are considered beneficial to human health as preventative agents. These results support the capacity of the ethyl acetate fraction of elephant apple bark to protect against oxidative stress [35].

### 3.2. Antimicrobial Activity

Antimicrobials—including antibiotics, antivirals, antifungals, and antiparasitics—are medicines of natural or synthetic origin that destroy or prevent the growth of microorganisms, thereby preventing and treating infections in humans and animals. Elephant apple demonstrated significant antimicrobial activity when it was explored for its antibacterial and antifungal activities. In a study, the antibacterial activities of elephant apple bark and fruit extracts were investigated, as well as the impact of inhibitory concentrations on the cell wall, nucleic acid leakage, and bacteria pathogenic genes. Using the agar dilution process, the bark and fruit extracts obtained by 70% aqueous acetone extraction had minimum inhibitory concentrations against different bacterial species in the range of 1250–5000 and 2000–10,000 mg/L, respectively, indicating that the bark extract had higher antibacterial activity. The ability of these extracts to trigger cell wall disintegration and genetic material leakage may be the driving force behind their antibacterial properties. Furthermore, the extracts inhibited pathogenic genes in the bacteria that were tested. Elephant apple bark and fruit aqueous acetonic extracts’ antimicrobial activity has been due to the presence of phenolic compounds [60]. These findings suggest that elephant apples have a high antimicrobial potential, making them useful in the treatment of microbial ailments including diarrhoea, dysentery, septicaemia, and skin problems.

### 3.3. Antidiabetic Activity

Diabetes is a progressive metabolic condition associated with increased blood sugar levels, which can cause permanent damage to the blood vessels, kidneys, eyes, heart, and nerves over time [61]. Diabetes affects nearly 422 million people globally; most of the affected belong to low-and middle-income countries, and it is directly responsible for 1.6 million deaths per year (WHO 2016). Elephant apple has been used to treat diabetes in Indian culture for decades. The methanolic leaf extract (50 percent aqueous) of elephant apple decreases rat digestive sucrase and maltase enzymatic activity by 40 percent and 56 percent, respectively [62]. When tested in vivo on type 1 and type 2 diabetic rats caused by STZ at doses of 200 and 400 g/kg body weight (bw), the ethyl acetate fraction prepared from elephant apple methanolic leaves extract showed reductions in blood glucose, triglyceride, and serum cholesterol levels after 21 days. The high-density lipoprotein cholesterol (HDL-C) level in the treated rats also improved in comparison with the diabetic control group [63].

A new compound derived from the alcoholic extract (AE) of elephant apple leaves, chromane 3,5,7-trihydroxy-2-(4-hydroxybenzyl)-chroman-4-one, showed strong antidiabetic activity. STZ (50 mg/kg) was given AE (100, 200, and 400 mg/kg) and chromane (5 and 10 mg/kg) to see if they influenced fasting blood glucose, lipid levels, and serum insulin for 21 days. Oral administration of chromane and AE lowered fasting blood glucose, oxidative stress, and lipid levels significantly as compared to glimepiride (10 mg/kg). According to the results, elephant-apple-isolated AE and chromane have antidiabetic efficacy and may be used as therapeutic agents to monitor a variety of pharmacological diabetes treatment goals [55]. These results also backed up elephant apple’s common use as a diabetes remedy by indigenous peoples.

### 3.4. Anticancer Activity

Cancer is a general term that refers to a group of diseases that originate in almost any organ or tissue of the body and spread to other organs when abnormal cells grow out of control, cross their usual boundaries, and invade other parts of the body [64]. Cancer is the second leading cause of death in the world, accounting for 9.6 million deaths (or one of every six deaths) (WHO 2018). A lot of evidence suggests that a variety of cell lines tested for the anticancer activity of bioactive compounds found in different parts of the elephant apple have strong anticancer activity. In vitro, the cytotoxic activity of elephant apple fruit [49] and leaves [65] methanolic extracts against carcinoma, leukaemia, and lymphoma cells was reported. Methanolic extract from the fruit prevented the development of the cancer cell lines HL60, U937, and K562 with IC_50_ of 297.69 ± 7.29, 328.80 ± 14.77, and 275.40 ± 8.49 µg/mL, respectively, of commonly used drugs Gleevec and Ara C [49]. A major bioactive compound, betulinic acid, isolated using silica gel column chromatography from the ethyl acetate fraction prepared from methanolic extracts of elephant apple fruit showed IC_50_ of 12.84 ± 1.23, 13.73 ± 0.89, and 15.27 ± 1.16 µg/mL in cell lines HL60, U937, and K562 [49]. Betulinic acid has been reported to be potently effective against a broad range of in vitro cancer cells, including therapy-resistant and refractory tumours, while in healthy cells, it is reported to be relatively non-toxic [66]. The findings demonstrated elephant apple fruit’s methanolic extract to possess significant anticancer (anti-leukemic) activity against cell lines HL60, U937, and K562, where betulinic acid is the active constituent responsible for anticancer activity and was present in relatively good quantities in the methanolic extract and its ethyl acetate fraction with an insignificant n-butanol fraction [49].

### 3.5. Anti-Inflammatory Activity

Inflammation is a defensive response involving blood vessels, immune cells, and molecular mediators that occurs when body tissues are exposed to harmful stimuli such as pathogens, damaged cells, or irritants. The methanol extracts obtained from elephant apple leaves, when studied for their anti-inflammatory activity, demonstrated significant (*p* < 0.01) anti-inflammatory activity in the paw oedema test caused by carrageenan and capillary permeability stimulated by acetic acid at 200 mg/kg and 400 mg/kg and also demonstrated substantial (*p* < 0.05) activity against acid-induced permeability at 100 mg/kg [18]. The chloroform fraction obtained from the methanolic extract of elephant apple leaves has an anti-inflammatory effect in the paw oedema caused by carrageenan at doses of 25, 50, and 75 mg/kg p.o (per os; by mouth or oral administration) relative to the standard drug diclofenac sodium (5 mg/kg bw, p.o). At higher doses of 75 mg/kg, the effect is more pronounced compared to lower doses. The extract, which depends on the dosage, decreases inflammation substantially (*p* < 0.01) [67]. Flavonoids and triterpenes identified in the preparatory phytochemical analysis are likely to contribute to the anti-inflammatory effect. Methanolic extracts of elephant apple leaves (200, 400, and 800 mg/kg) and their chloroform and hexane fractions (200 and 100 mg/kg) ameliorate the effects of experimental colitis induced by acetic acid in female Swiss albino mice (25–30 g). An enhanced macroscopic score, colonic myeloperoxidase (MPO), colon weight, malonaldehyde (MDA), and tumour necrosis factor (TNF-α) levels were triggered by the intra-rectal acetic acid instillation, and the levels of colonic catalayse (CAT), glutathione (GSH), and superoxide dismutase (SOD) significantly decreases. Therapy of methanolic extracts (800 mg/kg), chloroform (200 mg/kg), and hexane fractions (200 mg/kg) has significantly reduced the macroscopic score, MPO, colon weight, MDA, and TNF-α levels and gradually increased the levels of CAT, GSH, and SOD. The plant extract and its fractions were found to exhibit significant (*p* < 0.05) anti-inflammatory effects. The findings indicate that elephant apple improves experimental colitis by hindering proinflammatory mediators, such as TNF-α development [68]. These results promote the traditional use of elephant apple in inflammatory diseases.

### 3.6. Antidiarrhoeal Activity

Diarrhoea is a symptom of a bacterial, viral, or parasitic infection in the intestine, where an infection transmits from person to person as a result of bad hygiene and/or by contaminated water and food. Diarrhoeal disease is the second leading cause of death in children under the age of five, resulting in the deaths of approximately 0.525 million children per year (WHO 2017). A substantial amount of evidence has shown that the active components extracted from the leaves and fruits of the elephant apple possess significant antidiarrheal activity. The antidiarrheal activity of elephant apple leaves when monitored against castor-oil-induced mice in a study using aqueous and methanol extracts demonstrated a decreased total amount of faeces at doses of 200 and 400 mg/kg after 2 h of treatment [69]. The leaf and fruit polar extracts triggered the onset and reductions in the frequency of defecation in the administered mice. The extracts that were used with charcoal meal demonstrated that it has the potential to reduce GI tract motility [70]. Ethanolic extracts of fruit and leaves lowered the overall amount of wet faeces and gastrointestinal motility in castor-oil-induced diarrheal mice at doses of 200 and 400 mg/kg relative to loperamide (5 mg/kg) [70]. The antidiarrheal action and extension of onset might have been caused by the inhibition of inflammatory mediator release as well as the contribution to antidiarrheal activity by phytoconstituents such as flavonoids and tannins [18,69]. Plant ethanolic extracts were more potent against diarrhoea caused by castor oil than aqueous extracts. Moreover, ethanolic extracts significantly reduce diarrhoea induction time and faeces total weight. Hence, the findings demonstrated that these plant extracts are effective as antidiarrhoeal agents [71].

### 3.7. Analgesic Activity

Pain is associated with certain pathological disorders. The phenomenon of reducing unpleasant feelings dates back to the dawn of time. Analgesics are pain relievers that function selectively without suppressing nerve signals, substantially modifying sensory perception, or altering consciousness. Elephant apple, when examined for its potential analgesic action in animal models from the crude methanol extract of the leaves, displayed substantial writhing inhibition in acetic-acid-induced mice with an oral dose of 250 and 500 mg/kg body weight (*p* < 0.01) relative to normal diclofenac sodium dosages of 25 mg/kg body weight [72]. The methanolic leaf extracts were also shown to be significantly compared with standard medicines, such as indomethacin (20 mg/kg bw) and pentazocine (15 mg/kg bw), when the central or peripheral analgesic activity was screened with a hot plate, tail immersion, formalin-induced nociception models, and acetic-acid-induced writhing of mice at a dose of 400 mg/kg bw [73]. In another study, the methanolic leaf extracts, when screened for their central or peripheral analgesic activity employing the tail-flick method, recorded a significant rise in pain tolerance at doses of 100, 200, and 400 mg/kg body weight and exhibited a maximum of 46.0% inhibition (*p* < 0.001) of the writhing reaction significantly compared to the standard drug diclofenac sodium (78.50%) when assayed on acetic-acid-induced writhing models at a dose of 400 mg/kg. The findings of the analysis show that the plant has a high analgesic ability, which may be correlated to the suppression of the central pain receptor [74].

### 3.8. Anxiolytic Activity

Anxiolytics are used for anxiety relief before surgery, as hypnotics in the treatment of insomnia, for conscious sedation and short-term relief of anxiety symptoms, and for the diagnosis of anxiety disorders [75]. A substantial amount of evidence has shown that the active components obtained from the leaves and bark extract of elephant apple possess significant anxiolytic activity. The hydroethanolic extract obtained from elephant apple leaves (50,100 mg/kg and 200 mg/kg p.o) when screened for its putative anxiolytic-like activity against the reference drug diazepam (2 mg/kg i.p (i.p.: intraperitoneal) in mice using open field, elevated plus maze, hole board, and light/dark exploration models exhibited significant results at doses of 200 and 400 mg/kg, whereas doses of 100 mg/kg had no effect against experimentally induced anxiety in mice. The findings confirmed that the hydroethanolic extract of elephant apple leaves possesses significant anxiolytic activity, which seems to be a promising approach and thus can be considered an alternative solution to anxiety [76].

### 3.9. Anti-Arthritic Activity

Arthritis is a condition in which one or more joints swell and become tender. Joint pain and stiffness are the most common signs of arthritis, which usually intensify with age. Osteoarthritis and rheumatoid arthritis are the two most prevalent forms of arthritis, where osteoarthritis leads cartilage to deteriorate and rheumatoid arthritis causes the immune system to invade the joints, starting with the joint lining. The chloroform fraction obtained from the methanolic extract of elephant apple leaves (CFMEDI) demonstrated remarkable anti-arthritic activity. The anti-arthritic activity was conducted using Complete Freund’s adjuvant-induced arthritis model triggered by the intraplantar injection of 0.1 mL in the left hind paw of rats. The CFMEDI at doses of 25, 50, and 75 mg/kg p.o (by mouth) was used for treatment relative to the standard drug cyclophosphamide (100 mg/kg bw). In adjuvant-induced arthritis rats, the huge rise in leukocyte counts may be triggered by activation of the immune system against the attacking antigens, and the decrease in the respective groups treated with CFMEDI showed its immunomodulation effect. CFMEDI and the regular medication cyclophosphamide have considerably offset the erythrocyte sedimentation rate (ESR) count, which has increased substantially in the arthritic control group, thereby recovering to almost normal, demonstrating the essential role of CFMEDI in arthritic conditions [67].

### 3.10. Hepatoprotective Activity

The ability of a chemical compound to protect the liver from any damage caused by oxidative stress and free radicals is referred to as hepatoprotective activity. The liver plays an important role in metabolism, storage, secretion, and removal of endogenous and exogenous toxic substances in the human body, and damage to the liver in any condition can be of serious concern to human health. The ethanolic extract of elephant apple leaves (300 mg/kg bw) was screened for hepatoprotective activity against the standard drug silymarin (25 mg/kg bw) in carbon tetrachloride (2.5 mL/kg bw)-induced hepatotoxicity in albino rats. The study demonstrated a significant reduction (*p* < 0.001) in various biochemical parameters, such as serum glutamic oxaloacetate transaminase, serum glutamic pyruvate transaminase, serum alkaline phosphatase, total bilirubin, and lipid peroxidation. Histopathological analysis supported the findings and thus confirmed the extract to possess pronounced hepatoprotective activity [77].

### 3.11. Antimutagenic Activity

Mutagenicity is characterised as the development of irreversible transmissible modifications in the composition of cells or organisms’ genetic material. A single gene or a group of genes can be affected by these variations (mutations). Antimutagenic agents are substances that can be used to combat the effects of mutagens. Both natural and synthetic substances are included in this category. The antimutagenic activities of the bark and fruit extracts of elephant apple using 70% aqueous acetone extraction were investigated against sodium-azide-induced mutations in the *Salmonella typhimurium* strain TA-1531 and displayed strong antimutagenic activity in the Ames test at ≥ 1500 μg/plate, where bark extract showed significantly (*p* < 0.01) higher antimutagenic activity at 500 μg/plate compared to fruit extract [60].

### 3.12. Anthelmintic Activity

Anthelmintics are medications that fight parasitic worm infections (helminth). Over 1.5 billion individuals, or 24% of the global population, are plagued with helminth infections caused by soil (WHO 2020). The anthelmintic potential of a methanolic extract of elephant apple bark (25 mg/mL) was investigated and compared against the standard drug albendazole (10 mg/mL). The findings concluded that the methanolic extract of elephant apple bark can induce paralysis of the worms at 136 min and death at 176 min, while albendazole (a positive control) paralysed and killed the worms at 17.67 min and 48 min, respectively. The findings indicate moderate anthelmintic activity of elephant apple bark extract and thus recommend the bioassay extraction of active principles [78].

### 3.13. Immunostimulating Activity

Immunostimulants are compounds that activate the immune system’s defence mechanisms by triggering or increasing the function of its components, enabling any animal to cope with diseases. The elephant apple fruit methanolic (70%) extracts exhibited an enhanced production of polyclonal immunoglobulin M (IgM) by 90.72% in cultured BALB/c female mice spleen cells at a concentration of 200 µg/mL as compared to lipopolysaccharide (0.1 µg/mL). The findings concluded that the elephant apple has mild immunostimulating activity for IgM production through the stimulation of B-cells and can boost the immune system of the body [79].

### 3.14. Antiplasmodial Activity

Antiplasmodial activity refers to the ability of antiparasitic drugs to destroy parasites of the *Plasmodium* genus. There are over 200 species in the family, including malaria parasites. Nguyen-Pouplin et al., (2007) examined the antiplasmodial activity of elephant apple leaves’ ethanolic extract (80%) using cyclohexane fraction against the standard drug chloroquione, which exhibited 53% inhibition against the FcB1 strain of *Plasmodium falciparum* at a concentration of 10 mg/mL, thus confirming the extract of elephant apple leaves to possess potent anti-malarial activity [80].

**Table 5:** Pharmacological studies on elephant apple plant extracts.

Plant Part (s)	Solvent Extract	Functional Properties	Reference(s)
Fruit	Methanol, ethyl acetate, aqueous, and acetone	Antioxidant	[34,81]
Bark	Acetone, methanol, and ethyl acetate		[8,35,82]
Leaves	Methanol, petroleum ether, chloroform, aqueous, ethanol, and hexane		[55,68,83,84]
Leaves	Methanol, carbon tetrachloride, chloroform, and hexane	Antimicrobial	[19]
Bark	Methanol, hexane, dichloromethane, ethyl acetate, and acetone		[60,85]
Fruit	Acetone		[60]
Leaves	Methanol, and ethyl acetate	Antidiabetic	[42,55,62,63,86]
Fruit	Methanol, and ethyl acetate	Anticancer	[49]
Leaves	Methanol		[65]
Leaves	Methanol, chloroform, and hexane	Anti-inflammatory	[18,67,68]
Leaves	Aqueous, methanol, and ethanol	Antidiarroheal	[69,70,72]
Fruit	Ethanol		[70]
Leaves, Bark	Methanol	Analgesic	[8,72,73,74]
Leaves	Hydroethanolic	Anxiolytic	[76]
Bark	Methanol		[74]
Leaves	Methanol and chloroform	Anti-arthritic	[67]
Leaves	Ethanol	Hepatoprotective	[77]
Fruit and bark	Acetone	Antimutagenic	[60]
Bark	Methanol	Anthelmintic	[78]
Fruit	Methanol	Immunostimulating	[79]
Leaves	Ethanol and cyclohexane	Antiplasmodial	[80]

## 4. Traditional Uses and Therapeutic Importance of Elephant Apple

Elephant apple is a wild fruit with several nutritional and medicinal properties. This fruit is loved in many different ways, whether it is eaten raw or cooked, and it has an acidic flavour that makes it suited for a variety of dishes. Traditionally, it has been used to prepare delicious items such as juice, pickles, curry, jams, chutney, and jellies, adding to its versatility and culinary appeal. It is considered a potential source of nutrients and can be processed into value-added products for the market, such as squash and ready-to-serve beverages [14,87]. Beyond its culinary appeal, the fruit is believed to offer numerous health advantages. In various diseases, elephant apple has been found to possess strong therapeutic qualities. In conventional and pharmacological forms, different parts are used to treat illnesses and diseases. The whole elephant apple plant is conventionally used as an aphrodisiac for fever, and it encourages virility; its decoction can be used as a universal antidote. Elephant apple stem bark acts as an ingredient in sores due to mercury poisoning, chronic progredient sores, carbuncles, and as a cholera season prophylactic. The extract from the stem added to and around a spider bite wound helps kill the poison [23,88]. The mucilage of elephant apple fruit is used for hair care and baldness [89]. For preventing cancer and diarrhoea, blended juices of bark and leaves are used orally [18,69,90]. The bark and leaves are also used as purgative and astringent [24].

Elephant apple leaves and fruit are used for diseases such as fever, dysentery, constipation, and stomach ailments [4]. Juice, decoction, poultice, and the mucilage of elephant apples are used for the treatment of diarrhoea, diabetes, cancer, wounds, rheumatism, skin diseases, coughs, urinary problems, aches, hair loss, and fever. The fruit, leaves, and bark of the elephant apple can be used to treat skin problems like eczema, skin rashes, leukoderma, and skin itches [91]. The juices from elephant apple fruits, leaves, and bark are blended and used orally to reduce cancerous growths, especially gastric and breast cancers, and treat diarrhoea [92,93]. The fruit juice is cardiotonic; it promotes soothing during fevers and is also used for coughs [94,95]. The blended juice of sepals and fruit is used to treat diabetes [96,97]. The bark and roots of elephant apple have also been reported to be used as neutralisers for food poisoning [98]. In general, elephant apples contain a wide variety of compounds that address a wide range of health problems.

## 5. The Application of Elephant Apple as Pharmaceutical Excipients

A vast majority of studies have focused on the natural mucilaginous extract of elephant apple as a pharmaceutical excipient for drug delivery. A high swelling output with considerable mucous adhesive force was observed when characterised by Fourier Transformation Infrared Spectroscopy (FTIR), X-ray diffraction techniques (XRD), and Thermogravimetric Analysis (TGA) in the natural mucilaginous substances isolated from the seeds of elephant apple. The zeta potential of the mucilage was explored and found to increase the positive potential at −17.2. In vitro swelling and mucoadhesion characteristics were determined, and the mucilage with high swelling capacities was found to have an appreciable strength of mucoadhesives. In addition, analytical studies have shown that the mucilage is amorphous and has a mass loss event of one step. Therefore, elephant apples’ mucilage can be used to formulate various drug delivery systems as a natural mucoadhesive polymer [99].

For the preparation of felodipine mucoadhesive nasal gels, a known mucilaginous substance isolated from the fruit of elephant apple was assessed. It has proven to be a much stronger mucoadhesive agent than synthetic polymers, such as hydroxypropyl methylcellulose (HPMC). Gels made of elephant apple’s natural mucilaginous materials exhibited beneficial mucoadhesive properties, which have induced adherence to the nasal mucosa for a long time and thus enhanced the intranasal absorption by an excised goat nasal mucosa during the permeation test [100].

Polysaccharides obtained from elephant apples were evaluated for their ability to be used as polymers in drug delivery systems. In this regard, natural mucilaginous substances of elephant apple were obtained using the acetone precipitation method and subjected to identification and quantitative determination by carbazole and compatibility tests by FTIR. The physicochemical analysis of elephant apple mucilaginous substances showed a presence of pectin, while quantitative determination by carbazole also showed a presence of pectin, and the FTIR report shows its compatibility with pure pectin as well as Metformin HCl (the model drug), thus proving itself a promising polymer [101].

Pantoprazole is a drug used to treat gastric ulcers and gastroesophageal illness as a pump inhibitor of protons. In the gastrointestinal tract, pantoprazole must be absorbed, and enteric supply systems are necessary as it is unstable under acidic conditions. The mucilaginous extract obtained from elephant apple is used with sodium alginate by the ionotropic gelation technique and the Eudragit L100-55 coating to prepare microbeads loaded with pantoprazole. The spherical shape of the microbeads with adequate swelling, mucoadhesive properties, and acid resistance has been shown. This can be regarded as essential for its use, particularly for controlled release, in the delivery of mucoadhesive drugs [102].

The evaluation of the supply of low bioavailable drugs by buccal delivery with a naturally isolated mucoadhesive material from the fruit elephant apple has been reported to be a novel drug delivery system [103].

The pharmaceutical potential of *Dillenia indica* seed sap as a bioactive delivery system was explored in a study. *D. indica* mucilage-based beads containing vitamin E analogous (α-tocopherol acetate) in a core–shell arrangement were developed using alginate. The hybrid mucilage–alginate matrix demonstrated higher resistance to degradation during gastrointestinal phases and thermal treatments compared to traditional formulations. Furthermore, the beads exhibited good thermo-protective effects during high-temperature processing and efficiently released α-tocopherol acetate for absorption during in vitro digestion. These findings suggest that the facile synthesis of hybrid carriers from *D. indica* mucilage could offer promising applications for fruit waste in pharmaceutical contexts [104].

## 6. Concluding Remarks and Future

The review emphasised the potential bioactive compounds and the health benefits of elephant apple as an herbal medicine. In this review, we have discussed detailed pharmacology, chemical constituents, traditional uses of elephant apple, and its application in drug formulation. The available literature and reports indicate that elephant apple has adequate therapeutic potential and can be further investigated for chemical and pharmacological studies. In comparison to synthetic medicines, herbal medicines are considered beneficial due to the presence of numerous bioactive compounds without having adverse effects on human health. However, there have been only limited in-depth investigations that explored the extraction and identification of bioactive compounds from elephant apple. High-performance analytical studies are needed for the utilisation of functional ingredients extracted from underutilised fruits, particularly elephant apple. Altogether, innovative extraction strategies are needed to identify novel bioactive functional ingredients that may substantially reduce the risk of noncommunicable diseases.

## Figures and Tables

**Table 1:** Isolated phenolic compounds and flavonoids from different parts of elephant apple.

Isolated Compound	Plant Part(s)	Structure	Reference(s)
Kaempferide	Leaf	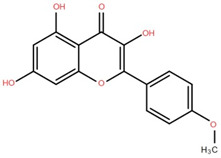	[39]
Kaempferide 3-O-diglucoside	Leaf	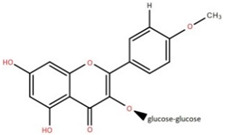	[39]
Quercetin	Leaf	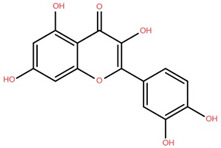	[39,42]
Rhamnetin	Leaf	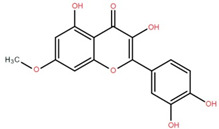	[39]
Dihydrokaempferide	Leaf	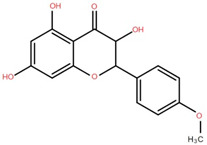	[39]
Dihydrokaempferide 7-diglucoside	Leaf	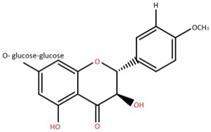	[39]
Leucocyanidin	Leaf	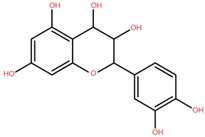	[39]
Naringenin 7-diglucoside	Leaf	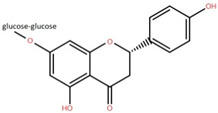	[39]
(+)-Dihydroxykaempferol	Twig	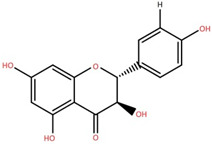	[36]
Gallic acid	Twig	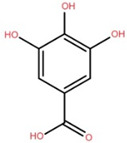	[36]
Isorhamnetin	Fruit, twig, and bark	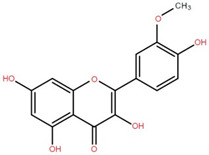	[36]
Dillenetin	Leaf	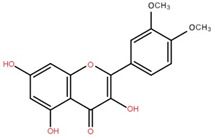	[43]
	Pericarp		[44]
Kaempferol	Pericarp, and twig	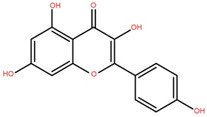	[36]
	Bark		[36,37]
Naringenin	Leaf	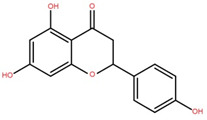	[39]
	Bark		[36]
Myricetin	Bark	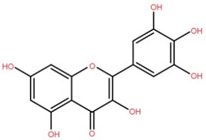	[25]
3′,5-Dihydroxy-4′,3- dimethoxy flavone-7-O-β-D-glucopyranoside	Bark	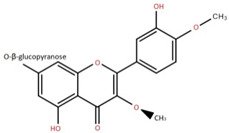	[45]
5,7-Dihydroxy-4′- methoxyflavone-3-O-β-D-glucopyranoside	Bark	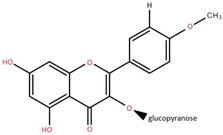	[45]
(+)-Dihydroisorhamnetin	Bark	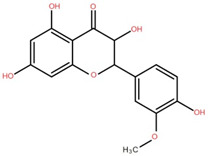	[44]
(+)-3′-Methoxydihydroquercetin	Bark	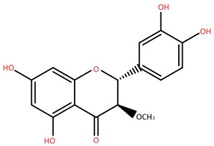	[36]
4,5,7,3′,4′-Pentahydroxy flavan-3-O-β-D-glucopyranoside	Bark	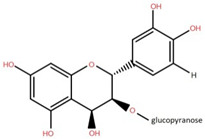	[45]
2-*O*-caffeoylhydroxycitric acid	Bark	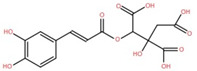	[35]
3,5-Dihydroxy-4-methoxybenzoic acid	Bark	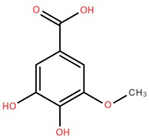	[35]
2-Caffeoylisocitric acid	Bark	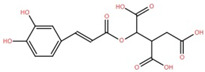	[35]
6,7,3′-Trihydroxy-2′,4′-dimethoxyisoflavan (bryaflavan)	Bark	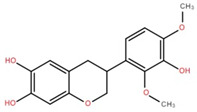	[35]
Amoradinin	Bark	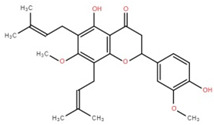	[35]
5,7-Dimethoxyapigenin	Bark	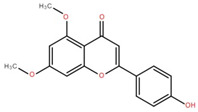	[35]
Formononetin 7-glucoronide	Bark	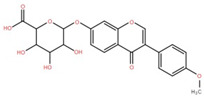	[35]
Mallotus B (isoallorottlerin)	Bark	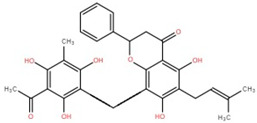	[35]

**Table 2:** Isolated triterpenoids and phytosteroids from different parts of elephant apple.

Isolated Compound	Plant Part(s)	Structure	Reference(s)
Betulinic acid	Bark	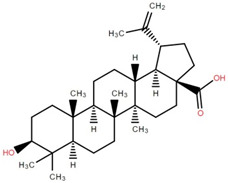	[15,25,51]
	Leaf		[42,43,48,52]
Lupeol	Bark	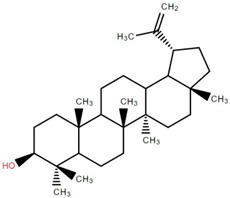	[15,25]
	Leaf		[52]
	Fruit		[50]
Betulin	Bark	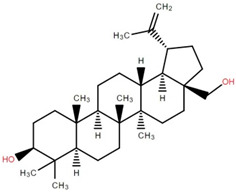	[25,51]
	Leaf		[52]
	Fruit		[50]
β-Sitosterol	Pericarp, twig, and bark	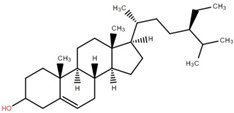	[36,44]
	Fruit		[50]
	Leaf		[42,43,48]
Stigmasterol	Bark	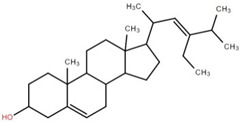	[15]
	Leaf		[42]
3β-Hydroxylupane-13β,28-lactone	Bark	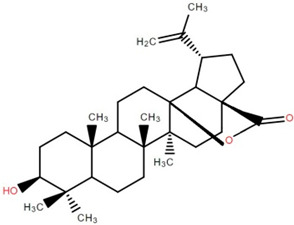	[25]
Betulinaldehyde	Bark	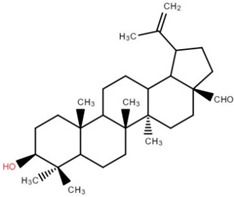	[15,25]
Nutriacholic acid	Bark	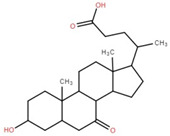	[35]
Cycloartenone	Leaf	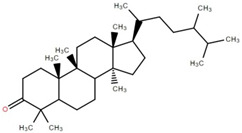	[48]
Stigmasteryl palmitate	Leaf	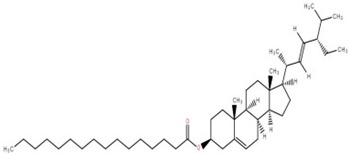	[42]

**Table 3:** Isolated alkaloids from bark of elephant apple.

Isolated Compound	Structure	Isolated Compound	Structure
Hydroxymethylserine	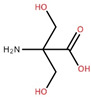	Triethanolamine	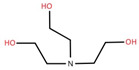
5-Acetyl-2,4-dimethylthiazole	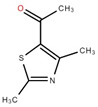	1,3-Bis(carbamoylcarbamoyl)urea	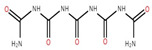
γ-Aminobutyric acid (GABA)	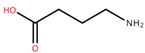	2-(Glucosyloxy) isobutyraldoxime	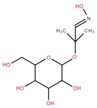
4-Methylthiazole-5-propionic acid	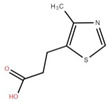	L-α-Aminosuberic acid	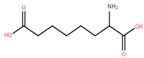
N-isopropylhydrazine carboxamide	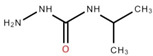	Linamarin	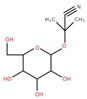
Dialanine	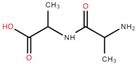	N-Acetyl-3,5,11,18-tetrahydroxyoctadecyl-2-amine	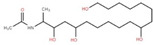

Source [35].

**Table 4:** Other compounds isolated from different parts of elephant apple.

Isolated Compound	Plant Part	Structure	Reference
3,5,7-Trihydroxy-2-(4-hydroxy-benzyl)-chroman-4-one	Leaf	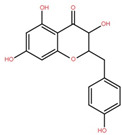	[55]
Malic acid	Fruit	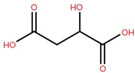	[56]
1,8-Dihydroxy-2-methylanthraquinone-3-O-β-D-glucopyranoside	Bark	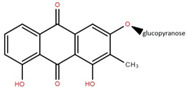	[45]
n-Hentriacontanol	Leaf	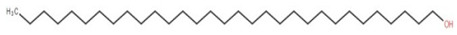	[48]
n-Heptacosan-7-one	Leaf	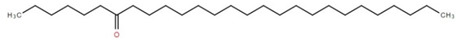	[42]
n-nonatriacontan-18-one	Leaf	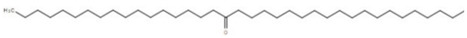	[42]
Ethyl maltol	Bark	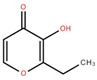	[35]
11-Dodecenoic acid	Bark	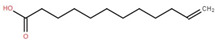	[35]
Glucose Na adduct	Bark	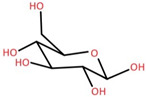	[35]

## Data Availability

Not applicable.
